# Multimodal analgesia strategies enhance postoperative recovery and mitigate inflammatory responses in women undergoing elective surgery for endometrial cancer: a retrospective cohort study

**DOI:** 10.3389/fonc.2026.1769801

**Published:** 2026-03-31

**Authors:** Yanhong Tang, Yumei He, Hai Yang, Hongyan Li, Anqiong Mao

**Affiliations:** 1 Department of Anesthesiology, The Affiliated Traditional Chinese Medicine Hospital, Southwest Medical University, Luzhou, Sichuan, China; 2 Department of Gynecology, The Affiliated Traditional Chinese Medicine Hospital, Southwest Medical University, Luzhou, Sichuan, China

**Keywords:** endometrial cancer, inflammation, multimodal analgesia, postoperative recovery, retrospective cohort study

## Abstract

**Background:**

Endometrial cancer is the most common gynecologic malignancy in developed countries, with rising global incidence attributed to population aging, obesity, and metabolic syndrome. Surgical resection is the cornerstone of treatment, but perioperative pain management remains challenging. Traditional opioid-centric regimens are effective for acute pain but are associated with adverse effects that can hinder recovery and potentially compromise oncologic outcomes. Multimodal analgesia, integrating non-opioid agents and regional techniques, is increasingly advocated but lacks disease-specific, large-scale comparative evidence in gynecologic oncology.

**Methods:**

A retrospective cohort study was conducted at a tertiary teaching hospital in Southwest China, including 650 women undergoing elective surgery for histologically confirmed endometrial cancer between January 2020 and January 2025. Patients were stratified into four groups according to perioperative analgesic regimens: (1) opioid-dominant IV PCA, (2) opioid-sparing plus NSAIDs, (3) epidural/regional adjunct, and (4) fully multimodal analgesia (regional, NSAIDs/acetaminophen, reduced opioids). Co-primary outcomes were prolonged hospitalization (>7 days) and any postoperative complication (Clavien–Dindo grade II or higher). Secondary endpoints included pain scores, incidence of postoperative nausea and vomiting (PONV), time to first ambulation/flatus, and perioperative immune-inflammatory markers (NLR, CRP). Multivariable logistic regression and linear mixed-effects models were used to adjust for potential confounders.

**Results:**

Baseline demographic and clinical characteristics were well balanced across groups. Patients receiving multimodal or regional-based regimens had significantly lower opioid consumption and mean pain scores on postoperative day 1 (NRS: 3.2 vs. 4.8, P<0.001) and lower PONV incidence (17.0% vs. 30.9%, P = 0.003) compared to opioid-dominant PCA. Multimodal and regional strategies were associated with earlier ambulation/flatus and shortened hospital stay (mean 6.5 vs. 8.2 days, P<0.001). The incidence of postoperative complications was lowest in the multimodal group (13.0% vs. 21.8%, P = 0.04). Postoperatively, NLR and CRP elevations were significantly attenuated in multimodal and regional groups (both P<0.001). Adjusted analyses confirmed that multimodal analgesia independently reduced the risk of prolonged hospitalization (OR 0.52, 95% CI 0.31–0.87, P = 0.013) and complications (OR 0.55, 95% CI 0.30–0.99, P = 0.048). Subgroup analyses demonstrated consistent benefit across age, BMI, surgical approach, tumor stage, and comorbidity strata.

**Conclusion:**

Comprehensive multimodal analgesia significantly reduces opioid consumption, improves pain control, accelerates postoperative recovery, and attenuates perioperative inflammatory responses in women undergoing surgery for endometrial cancer. These findings support the integration of multimodal analgesia into standard perioperative care protocols in gynecologic oncology, with the potential to enhance both clinical and biological outcomes. Prospective multicenter studies are warranted to validate these results and explore long-term oncologic implications.

## Introduction

Endometrial cancer represents one of the most prevalent malignancies of the female reproductive tract, with a global incidence that has steadily increased over recent decades, particularly in high-income and rapidly developing regions (1). This rise has been attributed not only to demographic transitions such as population aging, but also to the growing prevalence of obesity and metabolic syndrome, which are now acknowledged as principal risk factors in the pathogenesis of the disease (2). As a result, endometrial cancer imposes a substantial clinical and societal burden, manifesting in considerable morbidity, psychological distress, and significant healthcare expenditures. The impact of this disease is further magnified by the increasing trend in incidence and the persistent challenges in optimizing perioperative management and long-term survivorship (3).

Epidemiologically, endometrial cancer has become the most common gynecological cancer in developed countries, with its incidence continuing to rise globally (1). The contribution of obesity is particularly notable, as excess adiposity leads to chronic unopposed estrogen exposure and represents a central modifiable risk factor (4). Furthermore, advances in diagnostic modalities and increased awareness have facilitated earlier detection, resulting in a larger proportion of cases identified at a surgically resectable stage (5). Despite these advances, the disease remains a leading cause of cancer-associated morbidity among women, and variations in treatment strategies and outcomes persist across populations. The economic implications are equally significant, with the costs related to surgical care, adjuvant therapies, and management of perioperative complications imposing a growing strain on health systems (1).

Current standards of management for endometrial cancer are anchored in surgical intervention, typically total hysterectomy with or without lymphadenectomy, as the principal curative modality (3). However, the perioperative period is often marked by substantial physiological stress, pain, and risk of complications, all of which can adversely affect patient recovery trajectories. Pain control in the postoperative setting remains a persistent challenge, with traditional opioid-based regimens frequently employed as the mainstay of analgesia (6). While effective for acute pain relief, opioids are associated with a spectrum of undesirable effects—including nausea, vomiting, ileus, immunosuppression, and risk of dependence—that can hinder early mobilization, delay discharge, and potentially compromise oncologic outcomes (7). The recognition of these limitations has galvanized efforts to re-evaluate and refine perioperative pain management strategies, with a growing emphasis on approaches that mitigate opioid-related adverse effects while optimizing functional recovery.

In recent years, the concept of multimodal analgesia has garnered increasing attention as a cornerstone of enhanced recovery after surgery (ERAS) protocols in oncologic surgery (8). Multimodal analgesia involves the concurrent use of multiple analgesic agents and techniques—such as nonsteroidal anti-inflammatory drugs (NSAIDs), acetaminophen, regional nerve blocks, and adjuvant medications—with the aim of achieving synergistic pain control, reducing opioid consumption, and attenuating the surgical stress response (7, 9). Evidence from diverse surgical populations has demonstrated that multimodal regimens can improve postoperative pain scores, decrease the incidence of opioid-related side effects, and facilitate earlier mobilization and functional recovery (10). Nevertheless, there remains a paucity of high-quality, large-scale, and disease-specific investigations that systematically compare the impact of distinct analgesic strategies—including opioid-dominant, opioid-sparing, regional, and fully multimodal approaches—on recovery parameters and immune-inflammatory dynamics in endometrial cancer surgery.

Despite the theoretical and empirical advantages of multimodal analgesia, existing research has predominantly focused on single-agent interventions, procedure-specific cohorts, or surrogate endpoints, leaving critical gaps in understanding the comparative effectiveness of various perioperative pain management paradigms in the context of gynecologic oncology (6, 11). Specifically, there is limited evidence on how different analgesic strategies may influence not only immediate pain control but also broader recovery outcomes, complication rates, and perioperative immune-inflammatory responses, which are increasingly recognized as mediators of both short- and long-term prognosis in cancer patients (12). Furthermore, studies conducted in diverse geographic and healthcare settings remain scarce, underscoring the need for robust, contextually relevant data to inform best clinical practices and guideline development.

To address these knowledge gaps, the present study is designed as a retrospective cohort analysis embedded within a prospectively maintained electronic health record system at a major tertiary care center in southwestern China. The study will include a consecutive series of 650 patients who underwent elective surgical management for endometrial cancer between 2020 and 2025. Through rigorous case identification and stratification, patients will be categorized according to their perioperative analgesic regimen: opioid-based, opioid-sparing with NSAIDs, regional analgesia-assisted, and comprehensive multimodal analgesia. The study will employ advanced statistical methodologies, including multivariate regression and linear mixed-effects models, to adjust for confounders and assess independent associations between analgesic strategy and recovery outcomes.

The primary objective of this investigation is to systematically evaluate the differential effects of prevalent perioperative analgesia regimens on key clinical endpoints, such as length of postoperative hospitalization and incidence of major complications. Secondary endpoints will include patient-centered functional recovery, pain intensity scores, and perioperative immune-inflammatory biomarkers. By generating high-quality, real-world evidence on the comparative effectiveness of multimodal versus traditional analgesic approaches, this study seeks to advance the optimization of perioperative care in endometrial cancer and provide a scientific basis for evidence-informed clinical decision-making and guideline development in gynecologic oncology.

## Materials and methods

### Study design and population

**Figure 1:**
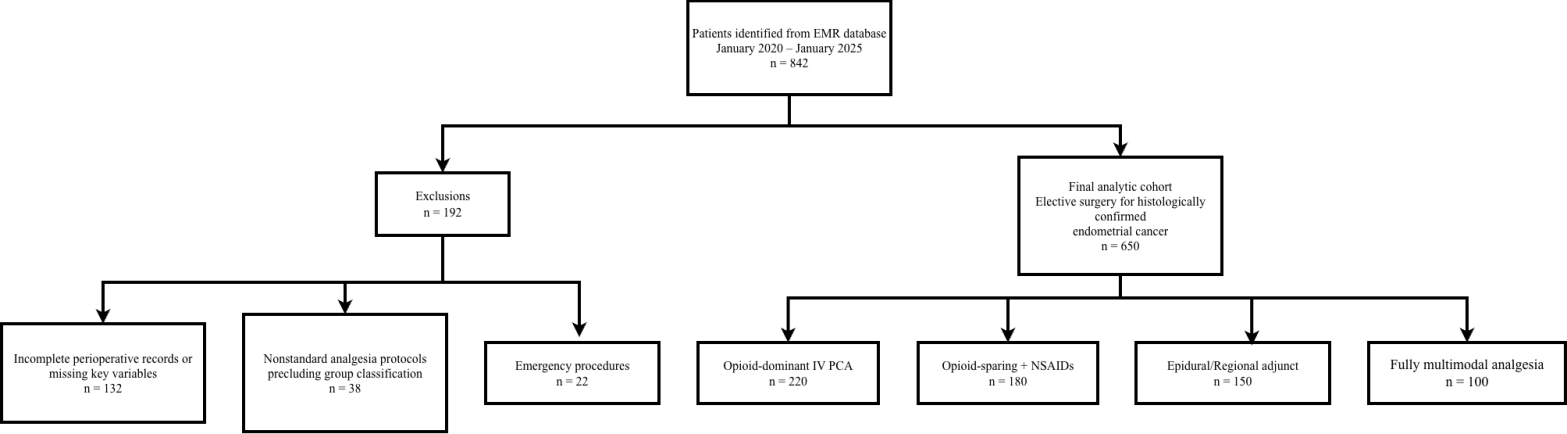
Study flow diagram of patient selection and cohort assembly. Patients were identified from the institutional electronic medical record (EMR) database between January 2020 and January 2025. Of 842 patients screened, 192 were excluded due to incomplete perioperative records or missing key variables (n = 132), nonstandard analgesia protocols precluding group classification (n = 38), or emergency procedures (n = 22). The final analytic cohort comprised 650 women undergoing elective surgery for histologically confirmed endometrial cancer. Patients were categorized according to their primary postoperative analgesia modality during the first 48 hours after surgery: opioid-dominant intravenous patient-controlled analgesia (IV PCA), opioid-sparing plus NSAIDs, epidural/regional adjunct, and fully multimodal analgesia.

This retrospective cohort study was conducted at a tertiary teaching hospital in Southwest China. In total, 842 patients were screened during the study period. We excluded 132 patients due to incomplete perioperative records or missing key variables required for the prespecified analyses (e.g., analgesia exposure classification and/or primary outcomes), 38 patients with nonstandard analgesia protocols that precluded group classification, and 22 emergency procedures. The final analytic cohort included 650 patients. The EMR query covered the entire study period (January 2020 to January 2025), and data extraction was performed using a standardized case-identification algorithm based on surgical scheduling records, pathology-confirmed diagnosis, and anesthesia charts. Details of patient screening and exclusions are provided in **Figure 1**. To minimize selection bias inherent to retrospective designs, data were extracted from a prospectively maintained electronic medical record (EMR) system. Two independent investigators performed the data extraction, and discrepancies were resolved by consensus. Cross-checking against original charts ensured completeness; among included patients, no variable used in the final models had more than 5% missingness.

### Data collection and variable definitions

Baseline demographic and clinical characteristics included age, body mass index (BMI), American Society of Anesthesiologists (ASA) physical status, diabetes mellitus, hypertension, and Charlson Comorbidity Index (CCI). Tumor-related variables were International Federation of Gynecology and Obstetrics (FIGO) stage and histologic grade. Operative variables encompassed surgical approach (open vs. minimally invasive), operative time, and estimated blood loss. Preoperative laboratory indices included hemoglobin concentration and neutrophil-to-lymphocyte ratio (NLR), while postoperative inflammatory markers comprised NLR and C-reactive protein (CRP) measured on postoperative day (POD) 1 and 3. Enhanced Recovery After Surgery (ERAS) adherence was assessed according to a standardized institutional checklist, with adherence ≥80% considered high.

### Analgesia group classification

Patients were categorized into four groups according to their primary analgesic regimen during the first 48 hours postoperatively. The opioid-dominant IV PCA group received intravenous patient-controlled analgesia (PCA) with opioids ≥70 morphine milligram equivalents (MME) without scheduled NSAIDs or acetaminophen. The opioid-sparing + NSAIDs group was characterized by opioid doses <70 MME combined with intravenous NSAIDs such as flurbiprofen or parecoxib and/or acetaminophen. The epidural/regional adjunct group included patients managed with continuous epidural analgesia or regional nerve blocks, such as transversus abdominis plane (TAP) or quadratus lumborum block (QLB), in conjunction with limited opioid use. Finally, the fully multimodal group received a comprehensive regimen combining regional block, NSAIDs/acetaminophen, and reduced-dose opioids. To minimize misclassification, only scheduled medication exposure was considered for group assignment, while single unscheduled rescue doses were excluded from classification. Group allocation reflected real-world clinical decision-making by anesthesiologists and surgeons, and baseline characteristics were well balanced across groups (**Table 1**).

**Table 1:** Baseline characteristics of patients with endometrial cancer undergoing surgery under general anesthesia, stratified by postoperative analgesia modality (n = 650).

Characteristics	Opioid-dominant IV PCA (n=220)	Opioid-sparing + NSAIDs (n=180)	Epidural/Regional adjunct (n=150)	Fully multimodal (n=100)	P-value
Age, years, mean ± SD	59.1 ± 9.7	58.8 ± 9.9	60.3 ± 9.4	58.7 ± 9.1	0.65
BMI, kg/m², mean ± SD	26.9 ± 3.8	27.2 ± 4.1	26.8 ± 3.7	27.0 ± 3.6	0.79
ASA class III–IV, n (%)	58 (26.4)	47 (26.1)	43 (28.7)	25 (25.0)	0.91
Diabetes mellitus, n (%)	64 (29.1)	53 (29.4)	41 (27.3)	26 (26.0)	0.85
Hypertension, n (%)	91 (41.4)	72 (40.0)	62 (41.3)	39 (39.0)	0.96
CCI ≥ 3, n (%)	42 (19.1)	32 (17.8)	29 (19.3)	18 (18.0)	0.95
Preoperative hemoglobin, g/dL, mean ± SD	11.8 ± 1.7	11.9 ± 1.6	11.7 ± 1.8	11.9 ± 1.7	0.81
Preoperative NLR, median (IQR)	2.9 (2.1–3.7)	2.8 (2.0–3.6)	2.9 (2.1–3.8)	2.7 (2.0–3.5)	0.72
FIGO stage III–IV, n (%)	84 (38.2)	65 (36.1)	52 (34.7)	34 (34.0)	0.77
High-grade histology, n (%)	96 (43.6)	77 (42.8)	64 (42.7)	40 (40.0)	0.94
Open surgery, n (%)	101 (45.9)	78 (43.3)	70 (46.7)	45 (45.0)	0.92
Operative time, min, mean ± SD	173.0 ± 41.5	169.5 ± 42.0	171.0 ± 39.6	170.2 ± 40.0	0.87
Estimated blood loss, mL, median (IQR)	300 (200–450)	280 (180–440)	290 (200–460)	295 (190–450)	0.78
ERAS adherence ≥ 80%, n (%)	147 (66.8)	124 (68.9)	104 (69.3)	71 (71.0)	0.87

All patients underwent surgery under general anesthesia. Analgesia modalities were defined according to the primary postoperative pain management strategy (see **Table 2** for details). Values are presented as mean ± standard deviation (SD), median (interquartile range, IQR), or number (%), as appropriate. ASA, American Society of Anesthesiologists; BMI, body mass index; CCI, Charlson Comorbidity Index; ERAS, Enhanced Recovery After Surgery; FIGO, International Federation of Gynecology and Obstetrics; NLR, neutrophil-to-lymphocyte ratio; PCA, patient-controlled analgesia.

### Outcomes

The study employed two co-primary outcomes. The follow-up window for all clinical outcomes was the index hospitalization, defined as the period from the end of surgery to hospital discharge. Prolonged hospitalization was defined as a postoperative length of stay (LOS) greater than 7 days, corresponding to the cohort median plus one day. Any postoperative complication was defined as Clavien–Dindo grade II or higher occurring during the index hospitalization, encompassing wound infection, urinary tract infection, pulmonary complications, and venous thromboembolism. Secondary outcomes included functional recovery indicators such as time to first ambulation and time to first flatus, as well as patient-centered measures including numeric rating scale (NRS, 0–10) pain scores on postoperative day (POD) 1 and the incidence of postoperative nausea and vomiting (PONV) within 48 hours. In addition, perioperative immune-inflammatory responses were assessed by serial changes in neutrophil-to-lymphocyte ratio (NLR) and C-reactive protein (CRP) measured at prespecified perioperative timepoints (preoperatively, POD1, and POD3). Clinically relevant thresholds were defined as NLR >5 and CRP >40 mg/L, values that reflect heightened systemic inflammation and perioperative immunosuppression.

### Statistical analysis

Continuous variables were assessed for normality using the Shapiro–Wilk test. Normally distributed data are presented as mean ± standard deviation (SD) and compared using one-way ANOVA, while non-normally distributed data are expressed as median (interquartile range, IQR) and compared using the Kruskal–Wallis test. Categorical variables are summarized as number (%) and compared using chi-square or Fisher’s exact test, as appropriate.

Multivariable logistic regression models were constructed to examine the independent association between analgesia modality and binary outcomes (prolonged LOS and any postoperative complication). Covariates were prespecified based on clinical relevance and prior literature, including age, BMI, ASA class, diabetes, hypertension, FIGO stage, surgical approach, operative time, preoperative NLR, and ERAS adherence. Model calibration was assessed with the Hosmer–Lemeshow goodness-of-fit test, with non-significant P values (>0.05) indicating good agreement between predicted and observed outcomes.

For repeated measures of NLR and CRP, linear mixed-effects models with random intercepts were applied to account for within-patient correlation across timepoints, adjusting for the same covariates. Subgroup and sensitivity analyses were conducted according to age (<65 vs. ≥65 years), BMI (<30 vs. ≥30 kg/m²), surgical approach (minimally invasive vs. open), FIGO stage (I–II vs. III–IV), and diabetes status. Interaction terms were tested to assess effect modification.

All statistical analyses were two-sided, with P <0.05 considered statistically significant. All clinical outcomes were analyzed within the index-hospitalization follow-up window (from surgery to discharge).Analyses were performed using SPSS (version 26.0; IBM Corp., Armonk, NY) and R (version 4.3.2; R Foundation for Statistical Computing, Vienna, Austria).

## Result

### Baseline characteristics

As shown in **Table 1**, baseline demographic, clinical, and surgical characteristics were well balanced across the four analgesia groups. The mean age ranged from 58.7 to 60.3 years, and the mean BMI from 26.8 to 27.2 kg/m², with no significant differences between groups. The proportions of patients with ASA class III–IV, diabetes mellitus, hypertension, or a Charlson Comorbidity Index (CCI) ≥3 were comparable. Preoperative laboratory indices, including hemoglobin levels and neutrophil-to-lymphocyte ratio (NLR), were also similar. With respect to tumor characteristics, the distribution of FIGO stage III–IV disease and high-grade histology did not differ significantly among groups. Intraoperative parameters, including surgical approach, operative time, and estimated blood loss, as well as ERAS adherence rates, were likewise evenly distributed. Collectively, these findings indicate that the study population was well matched at baseline, minimizing the likelihood of confounding due to imbalances in patient or disease characteristics (**Table 1**).

### Perioperative analgesia exposure and pain control

As summarized in **Table 2**, perioperative analgesia strategies differed substantially across groups. Patients receiving multimodal or regional-based regimens had significantly lower intraoperative and postoperative opioid requirements compared with those managed with opioid-dominant IV PCA (both P<0.001). The use of adjunctive agents was also consistent with treatment allocation: nearly all patients in the opioid-sparing and multimodal groups received NSAIDs and/or acetaminophen, whereas none in the opioid-dominant group did. Regional techniques were exclusively applied in the epidural/regional adjunct and multimodal groups, with epidural catheters being more frequent than TAP or QLB blocks. These variations in analgesic exposure translated into clinically relevant outcomes, as mean numeric rating scale (NRS) pain scores on POD1 progressively decreased from 4.8 in the opioid-dominant group to 3.2 in the multimodal group (P<0.001). Similarly, the incidence of postoperative nausea and vomiting (PONV) was highest in the opioid-dominant group (30.9%) and lowest in the multimodal group (17.0%; P = 0.003). Taken together, these findings confirm that multimodal and opioid-sparing strategies were associated with reduced opioid consumption and improved pain control, with fewer opioid-related adverse effects (**Table 2**).

**Table 2:** Perioperative analgesia exposure and pain control in patients with endometrial cancer (n = 650).

Analgesia exposure & outcomes	Opioid-dominant IV PCA (n=220)	Opioid-sparing + NSAIDs (n=180)	Epidural/Regional adjunct (n=150)	Fully multimodal (n=100)	P-value
Intraoperative opioid dose, MME, mean ± SD	145 ± 35	120 ± 32	115 ± 30	105 ± 28	<0.001
Postoperative opioid dose (48h), MME, mean ± SD	92 ± 20	55 ± 18	48 ± 16	40 ± 15	<0.001
NSAIDs administered, n (%)	0 (0.0)	168 (93.3)	128 (85.3)	95 (95.0)	<0.001
Acetaminophen administered, n (%)	0 (0.0)	124 (68.9)	102 (68.0)	79 (79.0)	<0.001
Regional block performed, n (%)	0 (0.0)	0 (0.0)	150 (100)	100 (100)	<0.001
– Epidural catheter, n (%)	—	—	110 (73.3)	65 (65.0)	—
– TAP/QLB block, n (%)	—	—	40 (26.7)	35 (35.0)	—
Mean NRS pain score POD1, mean ± SD	4.8 ± 1.0	3.9 ± 0.9	3.7 ± 0.8	3.2 ± 0.7	<0.001
PONV within 48h, n (%)	68 (30.9)	42 (23.3)	32 (21.3)	17 (17.0)	0.003

All patients underwent surgery under general anesthesia. Analgesia modalities were defined by scheduled exposure within the first 48 hours postoperatively: “Opioid-dominant IV PCA” = intravenous PCA with opioids ≥70 morphine milligram equivalents (MME) and no scheduled NSAIDs/acetaminophen; “Opioid-sparing + NSAIDs” = opioid dose <70 MME combined with intravenous NSAIDs (flurbiprofen, parecoxib) and/or acetaminophen; “Epidural/Regional adjunct” = continuous epidural analgesia or regional block (transversus abdominis plane [TAP] or quadratus lumborum block [QLB]) with limited opioids; “Fully multimodal” = combination of regional block plus NSAIDs/acetaminophen and reduced-dose opioids. Scheduled exposure was recorded, whereas single unscheduled rescue doses (if any) were not counted toward group assignment or medication exposure. MME, morphine milligram equivalent; NRS, numeric rating scale (0–10); NSAIDs, non-steroidal anti-inflammatory drugs; POD1, postoperative day 1; PONV, postoperative nausea and vomiting.

### Postoperative recovery outcomes and immune-inflammatory markers

As shown in **Table 3**, enhanced analgesic strategies were associated with accelerated postoperative recovery. Patients in the multimodal and regional adjunct groups ambulated and passed flatus significantly earlier than those in the opioid-dominant group (both P<0.001), and their mean hospital stay was shortened by nearly two days (6.5 vs. 8.2 days, P<0.001). Postoperative LOS was additionally summarized stratified by surgical approach (minimally invasive vs open), and the relative advantage of multimodal analgesia remained consistent within each stratum (**Supplementary Table 1**).The incidence of postoperative complications was also lower with multimodal analgesia (13.0%) compared with opioid-dominant PCA (21.8%, P = 0.04). Consistent with these clinical benefits, perioperative immune-inflammatory markers showed more favorable trajectories in patients receiving multimodal or regional-based regimens. Preoperative NLR and CRP were comparable across groups; however, postoperative elevations were significantly attenuated in multimodal and regional groups, with both NLR and CRP levels declining more rapidly by POD3 (all P<0.001). Collectively, these findings suggest that multimodal analgesia not only improves short-term recovery and reduces complications but also mitigates the perioperative inflammatory response (**Table 3**).

**Table 3:** Postoperative recovery outcomes and immune-inflammatory markers in patients with endometrial cancer (n = 650).

Outcomes & immune markers	Opioid-dominant IV PCA (n=220)	Opioid-sparing + NSAIDs (n=180)	Epidural/Regional adjunct (n=150)	Fully multimodal (n=100)	P-value
Time to first ambulation, h, mean ± SD	32.5 ± 6.8	27.8 ± 6.2	26.9 ± 6.0	24.7 ± 5.5	<0.001
Time to first flatus, h, mean ± SD	58.2 ± 12.4	51.7 ± 11.8	50.6 ± 11.5	48.1 ± 10.9	<0.001
Length of hospital stay, d, mean ± SD	8.2 ± 2.1	7.1 ± 1.9	6.9 ± 1.8	6.5 ± 1.7	<0.001
Any postoperative complication, n (%)	48 (21.8)	29 (16.1)	23 (15.3)	13 (13.0)	0.04
NLR, median (IQR) – preoperative	2.9 (2.1–3.7)	2.8 (2.0–3.6)	2.9 (2.1–3.8)	2.7 (2.0–3.5)	0.71
NLR, median (IQR) – POD1	5.2 (4.1–6.5)	4.4 (3.6–5.5)	4.2 (3.5–5.3)	3.9 (3.2–5.0)	<0.001
NLR, median (IQR) – POD3	3.9 (3.0–5.0)	3.3 (2.6–4.3)	3.2 (2.5–4.2)	2.9 (2.3–3.8)	<0.001
CRP, mg/L, mean ± SD – preoperative	7.8 ± 3.1	7.5 ± 3.0	7.6 ± 3.2	7.4 ± 3.0	0.83
CRP, mg/L, mean ± SD – POD1	42.6 ± 11.5	36.9 ± 10.8	35.7 ± 10.2	34.1 ± 9.8	<0.001
CRP, mg/L, mean ± SD – POD3	25.3 ± 8.4	21.5 ± 7.9	20.7 ± 7.6	19.4 ± 7.2	<0.001

All patients underwent surgery under general anesthesia. Analgesia modalities were defined as described in **Table 2**. Outcomes included postoperative recovery times and immune-inflammatory markers. Postoperative complications were defined as Clavien–Dindo grade II or higher, including wound infection, urinary tract infection, pulmonary complications, and venous thromboembolism. NLR, neutrophil-to-lymphocyte ratio (median [IQR]); CRP, C-reactive protein (mean ± SD); POD1 and POD3, postoperative day 1 and 3, respectively.

### Multivariable analysis of postoperative outcomes

After adjustment for demographic, clinical, and surgical covariates, analgesia modality remained an independent determinant of postoperative recovery (**Table 4**). Compared with the opioid-dominant PCA group, patients managed with fully multimodal analgesia had a 48% lower risk of prolonged hospitalization (>7 days; adjusted OR 0.52, 95% CI 0.31–0.87, P = 0.013). Opioid-sparing plus NSAIDs and epidural/regional adjunct regimens showed a similar trend toward reduced risk (OR 0.76 and 0.67, respectively), although neither reached statistical significance. A parallel pattern was observed for postoperative complications: multimodal analgesia was associated with a significantly lower risk (adjusted OR 0.55, 95% CI 0.30–0.99, P = 0.048), while the other regimens demonstrated non-significant reductions. Both models demonstrated good calibration by the Hosmer–Lemeshow test (P = 0.42 and 0.37, respectively), supporting the robustness of these findings. Taken together, these results underscore that a comprehensive multimodal analgesic approach provides the most consistent protection against adverse recovery outcomes.

**Table 4:** Multivariable logistic regression of analgesia modality and postoperative outcomes in patients with endometrial cancer (n = 650).

Outcomes & analgesia modality	Adjusted OR	95% CI	P-value
Length of stay >7 days*			
– Opioid-dominant IV PCA (Ref)	1.00	—	—
– Opioid-sparing + NSAIDs	0.76	0.51–1.14	0.19
– Epidural/Regional adjunct	0.67	0.44–1.02	0.06
– Fully multimodal	0.52	0.31–0.87	0.013
Hosmer–Lemeshow P	0.42	—	—
Any postoperative complication#			
– Opioid-dominant IV PCA (Ref)	1.00	—	—
– Opioid-sparing + NSAIDs	0.78	0.49–1.24	0.29
– Epidural/Regional adjunct	0.70	0.43–1.15	0.16
– Fully multimodal	0.55	0.30–0.99	0.048
Hosmer–Lemeshow P	0.37	—	—

Logistic regression models were adjusted for age, BMI, ASA physical status, diabetes, hypertension, FIGO stage, surgical approach (open vs minimally invasive), operative time, preoperative NLR, and ERAS adherence. Analgesia modalities were defined as in **Table 2**. *Length of stay (LOS) >7 days was defined as prolonged hospitalization, corresponding to the cohort median plus one day. #Postoperative complications were defined as Clavien–Dindo grade II or higher, including wound infection, pulmonary complications, urinary tract infection, and venous thromboembolism. The Hosmer–Lemeshow (HL) test was used to assess calibration, with a non-significant P value (>0.05) indicating good agreement between predicted probabilities and observed outcomes. OR, odds ratio; CI, confidence interval..

### Immune-inflammatory responses

**Figure 2:**
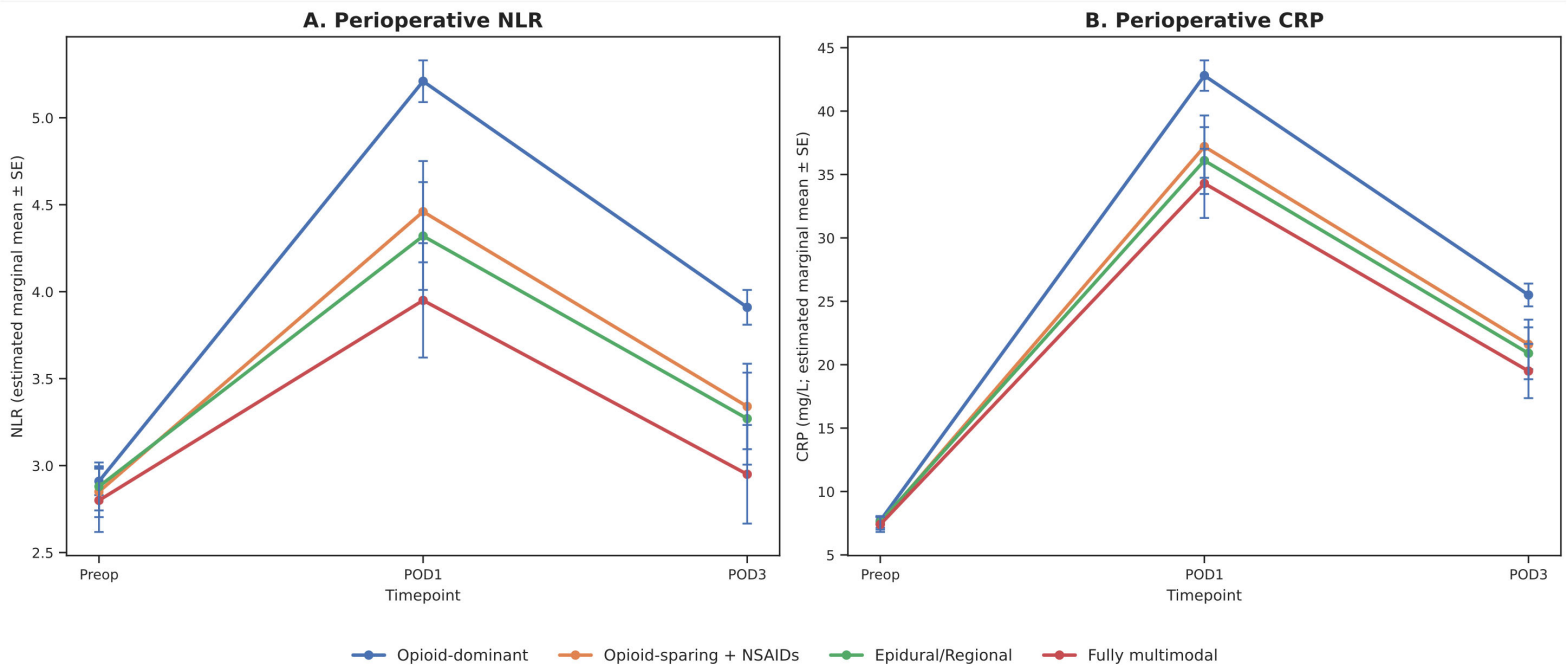
Perioperative immune-inflammatory trajectories by analgesia modality in patients with endometrial cancer. **(A)** Neutrophil-to-lymphocyte ratio (NLR) measured preoperatively and on postoperative day (POD) 1 and 3. **(B)** C-reactive protein (CRP) levels measured at the same timepoints. Values represent estimated marginal means (EMM) from linear mixed-effects models with standard errors (SE) indicated by error bars. Groups are defined in **Table 2**. NLR and CRP were comparable at baseline across analgesia groups, but postoperative elevations were significantly attenuated in the multimodal and regional adjunct groups compared with the opioid-dominant PCA group, with the greatest reductions observed in the fully multimodal group. Differences persisted through POD3, suggesting that multimodal analgesia mitigates perioperative inflammatory activation and may preserve immune competence. NLR, neutrophil-to-lymphocyte ratio; CRP, C-reactive protein; POD, postoperative day; PCA, patient-controlled analgesia.

As shown in **Table 5** and illustrated in **Figure 2**, baseline NLR and CRP values were comparable across the four analgesia groups, confirming balanced immune status before surgery. Postoperatively, however, distinct trajectories emerged. On POD1, the opioid-dominant group exhibited the greatest rise in both NLR and CRP, whereas patients managed with opioid-sparing, regional adjunct, or fully multimodal regimens showed significantly attenuated elevations. The multimodal group demonstrated the most pronounced reduction, with NLR values approximately 1.3 units lower and CRP levels about 8.5 mg/L lower than the opioid-dominant group (both P<0.001). By POD3, inflammatory markers had declined in all groups, but NLR and CRP remained significantly lower in the multimodal and regional groups compared with opioid-dominant PCA (all P<0.001). These consistent findings across timepoints, reflected in both the statistical models (**Table 5**) and visualized trends (**Figure 2**), indicate that multimodal analgesia not only improves clinical recovery but also exerts an immune-protective effect by mitigating perioperative inflammatory activation.

**Table 5:** Linear mixed-effects model analysis of perioperative analgesia modality and immune-inflammatory responses (n = 650).

Immune marker & timepoint	Opioid-dominant IV PCA (Ref, EMM ± SE)	Opioid-sparing + NSAIDs (Δ vs Ref, 95% CI)	Epidural/Regional adjunct (Δ vs Ref, 95% CI)	Fully multimodal (Δ vs Ref, 95% CI)	P for interaction
NLR					
Preoperative	2.91 ± 0.08	–0.06 (–0.24 to 0.12)	–0.03 (–0.22 to 0.16)	–0.11 (–0.32 to 0.10)	0.59
POD1	5.21 ± 0.12	–0.75 (–1.01 to –0.49)***	–0.89 (–1.17 to –0.61)***	–1.26 (–1.56 to –0.96)***	<0.001
POD3	3.91 ± 0.10	–0.57 (–0.79 to –0.35)***	–0.64 (–0.88 to –0.40)***	–0.96 (–1.22 to –0.70)***	<0.001
CRP, mg/L					
Preoperative	7.7 ± 0.3	–0.2 (–0.8 to 0.4)	–0.1 (–0.7 to 0.5)	–0.3 (–1.0 to 0.4)	0.77
POD1	42.8 ± 1.2	–5.6 (–7.7 to –3.5)***	–6.7 (–9.0 to –4.4)***	–8.5 (–10.9 to –6.1)***	<0.001
POD3	25.5 ± 0.9	–3.9 (–5.6 to –2.2)***	–4.6 (–6.4 to –2.8)***	–6.0 (–7.9 to –4.1)***	<0.001

Values are estimated marginal means (EMM ± standard error, SE) from linear mixed-effects models with random intercepts, adjusted for age, BMI, ASA class, diabetes, hypertension, FIGO stage, surgical approach, and ERAS adherence. Results are expressed as differences (Δ) compared with the opioid-dominant IV PCA group (reference), with 95% confidence intervals. Analgesia modalities were defined as in **Table 2**. NLR, neutrophil-to-lymphocyte ratio; CRP, C-reactive protein; POD, postoperative day. Clinically, NLR >5 and CRP >40 mg/L are considered thresholds of heightened inflammatory and immunosuppressive response. ***P <0.001 versus opioid-dominant IV PCA group.

### Subgroup and sensitivity analyses

Subgroup analyses further confirmed the robustness of the association between multimodal analgesia and reduced risk of prolonged hospitalization (**Table 6**). Across nearly all clinical strata, fully multimodal regimens were associated with lower odds of LOS >7 days compared with opioid-dominant PCA, with effect sizes ranging from a 39% to 61% relative reduction. The protective effect was statistically significant in younger patients (<65 years; adjusted OR 0.45, 95% CI 0.26–0.78), those with BMI <30 kg/m² (OR 0.47, 95% CI 0.28–0.80), patients undergoing minimally invasive surgery (OR 0.39, 95% CI 0.21–0.71), early-stage disease (FIGO I–II; OR 0.42, 95% CI 0.23–0.75), and those without diabetes (OR 0.46, 95% CI 0.27–0.79). Although similar trends were observed in older, obese, open-surgery, advanced-stage, and diabetic subgroups, these associations did not reach statistical significance. Importantly, none of the interaction terms were significant, indicating that the beneficial effect of multimodal analgesia was consistent across patient subgroups without evidence of effect modification. These findings support the generalizability and stability of the main results.

**Table 6:** Subgroup and sensitivity analyses of fully multimodal analgesia versus opioid-dominant IV PCA for prolonged length of stay (LOS >7 days) in patients with endometrial cancer (n = 650).

Subgroup	Opioid-dominant IV PCA (Ref)	Fully multimodal (Adjusted OR)	95% CI	P-value	P for interaction
Age					
<65 years (n=390)	1.00	0.45	0.26–0.78	0.004	
≥65 years (n=260)	1.00	0.61	0.34–1.09	0.095	0.21
BMI					
<30 kg/m² (n=470)	1.00	0.47	0.28–0.80	0.006	
≥30 kg/m² (n=180)	1.00	0.59	0.31–1.13	0.11	0.27
Surgical approach					
Minimally invasive (n=370)	1.00	0.39	0.21–0.71	0.002	
Open surgery (n=280)	1.00	0.64	0.36–1.13	0.12	0.18
FIGO stage					
I–II (n=380)	1.00	0.42	0.23–0.75	0.004	
III–IV (n=270)	1.00	0.61	0.35–1.06	0.081	0.24
Diabetes mellitus					
No (n=460)	1.00	0.46	0.27–0.79	0.005	
Yes (n=190)	1.00	0.66	0.36–1.21	0.18	0.22

Multivariable logistic regression models were adjusted for age, BMI, ASA physical status, hypertension, FIGO stage, surgical approach, operative time, preoperative NLR, and ERAS adherence, consistent with the main model in **Table 4**. Fully multimodal analgesia was compared with opioid-dominant IV PCA as the reference. Analgesia modalities were defined as in **Table 2**. LOS, length of stay; OR, odds ratio; CI, confidence interval. Interaction terms were tested for each subgroup; none were statistically significant, suggesting that the beneficial effect of multimodal analgesia on reducing prolonged LOS was consistent across patient subgroups. *P <0.05 was considered statistically significant.

## Discussion

Endometrial carcinoma represents one of the predominant malignancies affecting the female reproductive tract, with its incidence escalating in parallel with demographic shifts and lifestyle alterations globally. This neoplasm imposes profound physiological and psychological burdens on patients, compounded by substantial healthcare resource utilization. Surgical excision remains the cornerstone of curative intervention; however, perioperative management, particularly postoperative analgesia, plays a critical role in shaping recovery trajectories. Traditional opioid-centric analgesic regimens, while effective in mitigating nociceptive pain, are frequently accompanied by adverse sequelae including nausea, vomiting, immunosuppression, and delayed convalescence, which collectively hinder optimal postoperative rehabilitation. The imperative to refine analgesic strategies is underscored by the need to enhance early functional restoration and minimize complications, thereby improving overall patient outcomes and reducing societal costs.

This investigation systematically evaluates the impact of diverse perioperative analgesic protocols on postoperative recovery and inflammatory-immune responses in endometrial cancer patients undergoing elective surgery. By leveraging a substantial retrospective cohort with rigorous statistical adjustments, the study contrasts opioid-dominant, opioid-sparing combined with NSAIDs, regional block-assisted, and fully multimodal analgesia approaches. The comprehensive analysis elucidates the differential effects of these regimens on hospital stay duration, complication rates, pain control efficacy, and immunoinflammatory modulation. These findings not only corroborate the clinical superiority of multimodal analgesia in facilitating enhanced recovery but also set the stage for an in-depth discourse on the mechanistic interplay between analgesic modalities and immune-inflammatory dynamics in the postoperative milieu.

A critical prerequisite for attributing outcome differences to perioperative analgesic strategies is the baseline equivalence of clinical and demographic characteristics across study groups. In this study, the four analgesic cohorts demonstrated no significant difference in age, BMI, comorbidity burden, tumor staging, surgical modality, or intraoperative parameters, thereby minimizing potential confounding from patient- or procedure-related risk factors. This methodological rigor contrasts with many retrospective analyses of perioperative interventions, where imbalances in comorbidity profiles or operative complexity can obscure true treatment effects or lead to spurious associations (13). Notably, previous research on cancer surgical populations has identified age, metabolic comorbidities, and tumor burden as independent determinants of postoperative complication risk and recovery trajectories (14, 15). The present study’s thorough matching thus enhances the internal validity of intergroup comparisons and facilitates the isolation of analgesic modality as a primary variable of interest. Moreover, compared with large registry-based studies where residual confounding from unmeasured variables often remains a concern, the stringent baseline matching in this cohort aligns with best practices observed in randomized controlled trials and well-designed prospective cohorts (13). This rigorous approach additionally supports the external comparability of the findings, as the enrolled population’s characteristics reflect those reported in international cohorts of gynecologic oncology surgery, while acknowledging that regional differences in BMI or comorbidity prevalence may influence generalizability (15). In summary, the methodological emphasis on baseline group comparability ensures that subsequent analyses robustly reflect the independent effects of analgesic interventions.

The observed superiority of multimodal analgesia in optimizing postoperative pain control and minimizing opioid consumption is mechanistic ally grounded in its capacity for multi-target modulation of nociceptive pathways. Notably, our findings align with the Society of Onco-Anesthesia and Perioperative Care (SOAPC) consensus practice guideline, which advocates an opioid-sparing, multimodal strategy integrating regional/neuraxial techniques with scheduled non-opioid analgesics to reduce opioid-related adverse effects in oncologic surgery (16). By concurrently employing regional nerve blocks, NSAIDs or acetaminophen, and limited-dose opioids, this strategy achieves synergistic analgesia, thereby reducing the cumulative opioid requirement and consequent adverse effects. This mechanism has been substantiated in prior studies, which demonstrate that integrating non-opioid agents and regional techniques attenuates peripheral and central sensitization, dampens inflammatory cascades, and blunts opioid-induced side effects, including postoperative nausea and vomiting (PONV) and bowel dysfunction (17, 18). Notably, the reduction in PONV incidence in the multimodal cohort aligns with established pharmacological evidence that NSAIDs and regional blocks circumvent the emetogenic and gastrointestinal inhibitory effects intrinsic to opioid receptor activation (17). However, the specific analgesic combinations employed and their relative contributions to outcome heterogeneity remain a subject of ongoing investigation, with some reports suggesting that the choice and dosing of adjunctive agents may further modulate efficacy and tolerability (17). Compared to regimens relying predominantly on systemic opioids, the observed multimodal approach offers a mechanistically robust and clinically validated pathway to both superior pain control and reduction of opioid-related morbidity.

A key finding in this analysis is the differential impact of pharmacologic regimens on postoperative pain scores and opioid-related side effects, particularly PONV. Mechanistically, NSAIDs and regional anesthetic techniques exert their effects by inhibiting cyclooxygenase-mediated prostaglandin synthesis and blocking afferent neural transmission, respectively, thereby providing analgesia largely independent of central opioid receptors (17, 18). This contrasts with opioid monotherapy, which, while effective for acute nociception, is limited by a dose-dependent risk of emesis, sedation, and ileus due to its widespread action on central and enteric opioid receptors. Recent comparative studies corroborate that multimodal approaches incorporating NSAIDs or acetaminophen, and especially regional blocks, consistently yield lower pain scores, faster gastrointestinal recovery, and fewer emetogenic events than opioid-centric regimens (17). Intriguingly, while some trials report variable magnitudes of benefit depending on NSAID subtype or block technique, the overarching mechanistic principle of opioid-sparing analgesia remains consistent across surgical domains (17). Therefore, the findings of reduced pain and side effect burden in the multimodal and regional groups are biologically plausible and reflect a convergence of anti-inflammatory and neural blockade mechanisms that collectively optimize postoperative recovery.

The distinct side-effect profile associated with analgesic strategies is most evident in the frequency and severity of opioid-induced adverse events. The present data demonstrate that groups employing multimodal or regional techniques exhibit markedly lower rates of PONV and related opioid sequelae compared to those managed primarily with systemic opioids. This observation is mechanistically supported by the pharmacodynamics of non-opioid analgesics and regional anesthesia, which lack the central emetic and gastrointestinal inhibitory effects characteristic of mu-opioid receptor agonists (19, 20). Notably, while the risk of PONV is multifactorial and influenced by patient susceptibility, surgical factors, and adjunct medications, the proportional reduction achieved through opioid minimization has been consistently documented across both oncologic and non-oncologic surgical populations (19). Furthermore, recent reviews emphasize that the integration of regional techniques not only reduces systemic drug exposure but may also facilitate earlier ambulation and gastrointestinal recovery, indirectly mitigating other morbidity domains (18). The present results thus reinforce the mechanistic rationale and clinical benefit of opioid-sparing strategies for minimizing perioperative side effects.

Turning to short-term recovery endpoints, the acceleration of gastrointestinal function return and reduction in postoperative complication rates observed in the multimodal group can be attributed to both direct and indirect effects of opioid minimization and enhanced pain control. Mechanistically, opioids delay gastric emptying and impair enteric neural activity, whereas NSAIDs and regional blocks do not share this liability and may even foster pro-motility effects via anti-inflammatory actions (17, 18). Comparative analyses in other surgical cohorts confirm that multimodal strategies are independently associated with shorter times to ambulation, reduced ileus duration, and lower incidence of pulmonary and gastrointestinal complications (17). Moreover, multivariable modeling in recent studies validates that the observed reduction in overall morbidity is not solely a function of pain relief, but also reflects broader systemic effects on stress and inflammatory responses (18). These findings collectively indicate that the observed improvement in early recovery endpoints is a direct consequence of the mechanistic advantages conferred by opioid-sparing, multi-agent analgesic regimens.

The modulation of postoperative inflammation as reflected by biomarkers such as neutrophil-to-lymphocyte ratio (NLR) and C-reactive protein (CRP) is a noteworthy mechanistic outcome of analgesic strategy selection. Non-opioid agents, particularly NSAIDs, exert anti-inflammatory effects via inhibition of prostaglandin synthesis and attenuation of cytokine release, while regional blocks reduce local and systemic inflammatory signaling by limiting afferent nociceptive input (21). In contrast, opioid-dominant regimens do not directly target inflammatory cascades and may even impair postoperative immune competence through central and peripheral immunomodulatory effects (21). Prior studies have established that lower perioperative NLR and CRP are predictive of improved recovery and may correlate with reduced risk of infection and tumor progression in oncology patients (21). The current findings, demonstrating the lowest biomarker elevations in the multimodal and regional cohorts, are thus consistent with the hypothesized interplay between analgesic modality, inflammation, and immune homeostasis. Notably, while the precise prognostic value of postoperative NLR and CRP remains the subject of ongoing research, their attenuation via targeted analgesic strategies represents a plausible biological mechanism for improved clinical outcomes.

The application of robust statistical methodologies, including multivariable regression and mixed-effects models, is fundamental to the credibility of observed associations between analgesic strategies and clinical outcomes. Advanced modeling techniques facilitate the adjustment for known and unknown confounders and enable more precise estimation of independent treatment effects (22, 23). In contrast to univariate or inadequately adjusted analyses, which may be prone to overestimating intervention benefits due to residual confounding, the methodological approach adopted here aligns with contemporary standards for observational and quasi-experimental research (23). Recent literature underscores the importance of model choice, covariate selection, and interaction testing in ensuring the generalizability and reproducibility of perioperative outcome studies (22). The present study’s analytic framework, therefore, not only strengthens the internal validity of its findings but sets a methodological benchmark for future investigations in this domain.

Finally, the stability of multimodal analgesia’s benefit across diverse risk subgroups—stratified by age, BMI, surgical technique, tumor stage, and comorbidities—suggests a mechanistically robust and broadly applicable effect. Prior work in cardiovascular and oncologic surgery has highlighted the potential for differential intervention efficacy in distinct patient strata, often mediated by variations in baseline inflammatory activity, pharmacokinetics, or comorbidity burden (24, 25). However, the absence of significant interaction effects in this analysis parallels emerging evidence that multimodal, opioid-sparing strategies confer consistent benefit irrespective of baseline risk factor profile, likely owing to their multi-pronged mechanisms of action that address both nociceptive and systemic stress pathways (24). This finding supports the paradigm of individualized yet broadly deployable perioperative pain management.

This study possesses several notable limitations that warrant careful consideration. First, despite the use of a prospectively maintained electronic medical record system and rigorous data extraction protocols, the retrospective cohort design remains susceptible to unmeasured confounders and potential misclassification of exposure. The reliance on scheduled medication records for analgesia group assignment, although minimizing overt misclassification, may still overlook subtle variations in perioperative pain management practices or patient adherence, which could influence both primary and secondary outcomes. Second, the study was conducted at a single tertiary center with established ERAS protocols and high institutional adherence rates, potentially limiting the external validity and generalizability of the findings to broader clinical settings, especially those with differing resource availability, pain management preferences, or patient populations. Third, because standardized post-discharge follow-up (e.g., 30-day complications or readmissions) was not consistently available in the EMR for all patients, our analyses were limited to in-hospital outcomes, which may underestimate events occurring after discharge. These factors may restrict the applicability of the results to centers without comparable perioperative infrastructure or expertise. Taken together, our real-world evidence supports the practical implementation of guideline-concordant multimodal opioid-sparing pathways in gynecologic oncology, while underscoring the need for prospective multicenter studies to evaluate longer-term outcomes.

In summary, this investigation demonstrates that comprehensive multimodal analgesia confers significant benefits in reducing opioid consumption, enhancing postoperative recovery, and attenuating perioperative inflammatory responses among women undergoing surgery for endometrial cancer. The robust associations observed across multiple clinical and biochemical endpoints highlight the potential of multimodal strategies to improve both patient-centered and biological outcomes. Future research should prioritize prospective, multicenter trials to validate these findings in diverse patient populations and health care environments, while also elucidating the mechanistic pathways underlying immune modulation. The current results support the integration of multimodal analgesia as a standard component of perioperative care, with the potential to advance both immediate recovery and longer-term oncologic outcomes.

## Data Availability

The original contributions presented in the study are included in the article/**Supplementary Material**. Further inquiries can be directed to the corresponding authors.
