# Supplementary material for: Multimodal analgesia strategies enhance postoperative recovery and mitigate inflammatory responses in women undergoing elective surgery for endometrial cancer: a retrospective cohort study
Source: Front Oncol. 2026 Mar 31;16:1769801. doi: 10.3389/fonc.2026.1769801 (PMC13076245; doi:10.3389/fonc.2026.1769801)
Supplement: Supplementary file 1 [file Table1.docx]

**Supplementary Table S1.** Postoperative length of stay (LOS) stratified by surgical approach and analgesia modality (n = 650)

| **Surgical approach** | **Analgesia modality** | **n** | **LOS, mean ± SD (days)** |
| --- | --- | --- | --- |
| **Minimally invasive (n=356)** | Opioid-dominant IV PCA | 119 | 7.4 ± 1.8 |
|  | Opioid-sparing + NSAIDs | 102 | 6.6 ± 1.6 |
|  | Epidural/Regional adjunct | 80 | 6.3 ± 1.5 |
|  | Fully multimodal | 55 | 5.9 ± 1.4 |
| **Open surgery (n=294)** | Opioid-dominant IV PCA | 101 | 9.1 ± 2.0 |
|  | Opioid-sparing + NSAIDs | 78 | 8.0 ± 1.9 |
|  | Epidural/Regional adjunct | 70 | 7.8 ± 1.8 |
|  | Fully multimodal | 45 | 7.3 ± 1.7 |

**Notes:** Postoperative length of stay (LOS) was calculated from the end of surgery to hospital discharge during the index hospitalization. Values are presented as mean ± standard deviation (SD). Surgical approach was categorized as minimally invasive (laparoscopic or robotic) or open laparotomy. Analgesia modalities were defined as described in Table 2.
